# Lacrimal drainage system stenosis associated with Trastuzumab emtansine (Kadcyla®, T-DM1) administration: a case report

**DOI:** 10.1186/s12885-019-5986-5

**Published:** 2019-08-06

**Authors:** Chung Young Kim, Namju Kim, Ho-Kyung Choung, Sang In Khwarg

**Affiliations:** 1 Department of Ophthalmology, College of Medicine, Seoul National University, Seoul, South Korea; 2 Department of Ophthalmology, Seoul National University Hospital, Seoul, South Korea; 3 Department of Ophthalmology, Seoul National University Bundang Hospital, Seongnam-si, Gyeonggi-do South Korea; 4 Department of Ophthalmology, Seoul National University Boramae Hospital, Seoul, South Korea

**Keywords:** Trastuzumab emtansine, Kadcyla, Breast cancer, Nasolacrimal duct stenosis, Epiphora

## Abstract

**Background:**

Trastuzumab emtansine (Kadcyla®, T-DM1) is an antibody-drug conjugate used to treat HER2 (human epidermal growth factor receptor 2) overexpressing metastatic breast cancer. In this report, we present the first case of lacrimal drainage system stenosis identified after T-DM1 administration, and its successful treatment with a topical steroid.

**Case presentation:**

A 36-year-old female with metastatic breast cancer was referred for excessive tearing of both eyes. She previously underwent mastectomy and was treated with multiple anti-cancer regimens. However, metastases to liver and bone were identified and T-DM1 was administered. After 2 months, epiphora developed in both eyes and the patient was referred for ophthalmologic examination. The height of the tear meniscus was increased. The fluorescein dye disappearance test (FDDT) showed a delayed clearance in both eyes. Diagnostic lacrimal probing demonstrated a lower distal canalicular stenosis in both eyes. Dacryocystography indicated multiple focal narrowing of nasolacrimal duct in the right eye and diffused narrowing of nasolacrimal duct in the left eye. Topical eyedrop of tobramycin 0.3% and dexamethasone 0.1% were prescribed four times a day. After 2 months of treatment, the patient reported relief from epiphora, and the height of tear meniscus was normalized in both eyes.

**Conclusion:**

T-DM1 administration in breast cancer treatment can induce lacrimal drainage system stenosis, which can be treated effectively with a topical steroid.

## Background

Chemotherapeutic agents such as 5-fluorouracil, docetaxel, and S-1 induce nasolacrimal duct obstruction or stenosis [1]. However, the ocular adverse effects of targeted agents that are increasingly used in anti-cancer treatment are relatively unknown.

Trastuzumab emtansine (Kadcyla®, T-DM1) is an antibody-drug conjugate consisting of trastuzumab (targeting human epidermal growth factor receptor 2 (HER2)) and emtansine (microtubule-inhibitory agent, DM-1). It selectively delivers DM-1 to HER2 overexpressing tumor cells resulting in prolonged survival of patients with metastatic breast cancer, with reduced toxicity profile [2]. The main adverse effects are non-ocular; however, grade 1–2 ocular side effects such as conjunctivitis, swollen tear duct, increased lacrimation were reported in phase I/II clinical trials [3, 4]. Two case reports with corneal lesion induced by T-DM1 were published [5, 6], but no reports involving lacrimal drainage system have been presented so far. Herein, we report a case of lacrimal drainage system stenosis after administration of T-DM1 in advanced breast cancer.

## Case presentation

**Fig. 1:**
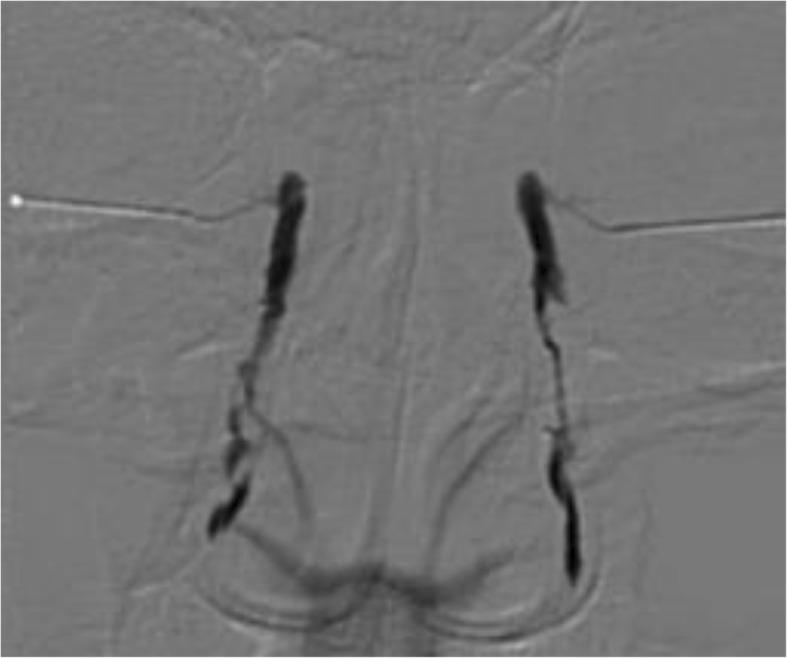
Dacryocystographic image of the patient showing multiple focal narrowing of nasolacrimal duct in right eye and diffuse narrowing of nasolacrimal duct in left eye

**Fig. 2:**
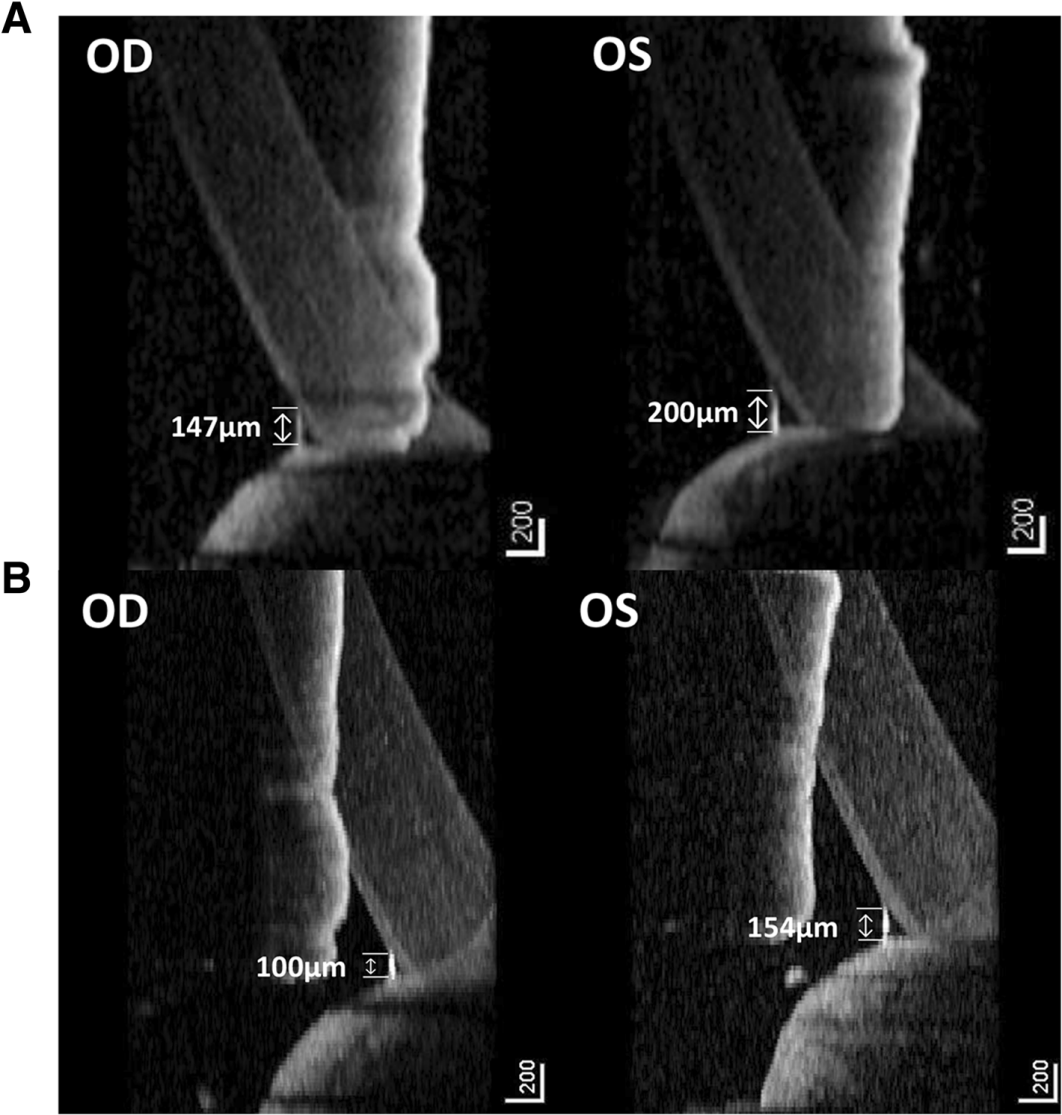
Anterior segment optical coherence tomography showing increased height of tear meniscus on her first visit (OD 147 μm, OS 200 μm) in (**a**) and decreased height of tear meniscus on follow up at 3 months (OD 100 μm, OS 154 μm) in (**b**)

A 36-year-old female with metastatic breast cancer presented with complaints of epiphora involving both eyes. She previously underwent skin-sparing mastectomy and was treated with anti-cancer regimens including docetaxel/doxorubicin/cyclophosphamide combinations and trastuzumab monotherapy. After local skin recurrence, the regimen was changed to vinorelbine plus epirubicin followed by a combination of capecitabine and lapatinib for 39 months. New metastases to liver and bone were found and T-DM1 was started. Two months after T-DM1 administration, epiphora developed in both eyes and the patient was referred for lacrimal system evaluation. The patient’s uncorrected visual acuity was 20/10 in the right eye and 20/13 in the left eye. The height of the tear meniscus was increased. The fluorescein dye disappearance test (FDDT) showed a delayed clearance in both eyes. The diagnostic lacrimal probing demonstrated a lower distal canalicular stenosis in both eyes. Dacryocystography showed multiple focal narrowing of nasolacrimal duct in the right eye and a diffuse narrowing of nasolacrimal duct in the left eye (Fig. 1). Topical tobramycin 0.3% plus dexamethasone 0.1% was administered four times a day during a month. After a month-long treatment, the height of tear meniscus was normalized in the right eye, but was slightly elevated in the left eye. Therefore, a continuous use of the eyedrop was recommended. After 2 months, she reported relief from epiphora and the height of the tear meniscus was normalized in both eyes (Fig. 2) The FDDT also revealed effective clearance in both eyes.

## Discussion and conclusions

Unlike traditional chemotherapy, targeted therapy selectively blocks the growth of cancer cells rather than attacking rapidly dividing cells. Ocular side effects involving the lacrimal drainage system associated with various chemotherapies are well documented; however, little is known about targeted therapies.

The adverse effects of antibody-drug conjugates need to be traced to the specific individual components, in this case, emtansine and trastuzumab. Emtansine, a derivative of maytansine, is attached to a monoclonal antibody and enters the cells to inhibit microtubule polymerization [7]. Ocular adverse effects of maytansine family members have yet to be reported; however, the nasolacrimal adverse effects of anti-neoplastic agents with similar mechanism of action, such as docetaxel, are well documented [8]. Docetaxel causes epiphora and induces stenosis of lacrimal drainage system [9] and stromal fibrosis in the mucosal lining of the affected lacrimal drainage system, and it has been confirmed by histopathologic study [10]. Because emtansine and docetaxel share a similar mechanism of action, it can be assumed that canalicular and nasolacrimal stenosis in our patient might be due to the adverse effect of emtansine. Two corneal lesions were reported as ocular adverse effects of T-DM1 administration [5, 6] based on the expression of HER2 in corneal epithelial cells and its susceptibility to trastuzumab harboring agents. In literature, two studies have reported HER2 overexpression in adenocarcinoma of lacrimal sac and nasolacrimal duct [11, 12], but not in normal nasolacrimal epithelium. If HER2 is also expressed in the normal nasolacrimal epithelium, nasolacrimal stenosis in our case can be explained in part as an effect of trastuzumab. Future study on the expression of HER2 in normal nasolacrimal system is required to elucidate the pathophysiology [13].

T-DM1 shows a higher efficacy compared with trastuzumab alone due to its dual effect on HER2 signaling and cytotoxicity, also resulting in a higher intensity of adverse effects. Ocular adverse effects were reported in 31.3% of T-DM1 [4] compared with 2.5% following trastuzumab monotherapy [14]. However, the comprehensive clinical manifestations and prevalence of nasolacrimal adverse effects have yet to be reported. Severe epiphora causes not only patient discomfort but also visual dysfunction. Future study of its adverse effects on the nasolacrimal system is certainly required.

Instillation of a topical steroid (combination of tobramycin and dexamethasone) was effective in providing symptomatic relief and quantitative decrease of tear meniscus in this case. In literature, a study reported a 61% efficacy with topical steroids in nasolacrimal duct obstruction [15]. Inflammation and edema of lacrimal drainage are the main pathologic findings in the early phase of lacrimal drainage system stenosis [16], which can be reversed by anti-inflammatory treatments such as topical steroids. Our group previously reported the effectiveness of topical steroid instillation in patients with recent lacrimal drainage stenosis and cumulative improvement was found in 51% of the 108 eyes (63% of cases with idiopathic nasolacrimal drainage stenosis, 100% of patients treated with docetaxel, and 43% of S-1-treated patients) [17].

In our patient, the duration of epiphora was relatively short and the onset was bilateral, simultaneous and concurrent with gradual exacerbation of symptoms after 2 months of T-DM1 treatment. Based on the previous findings mentioned above, it is speculated that the topical steroid was effective in controlling the inflammation and edema of nasolacrimal epithelium resulting in symptom relief and decrease in tear meniscus. It is possible that the epiphora and lacrimal drainage stenosis in this case were coincidental findings induced by other etiologies and not T-DM1. However, no other possible etiologies such as infection, endogenous or exogenous inflammation, internal or external mechanical problem or trauma were detected in previous history, symptoms or signs of the physical examination or in radiological tests and repeated blood labs. Although a direct cause-effect relationship was not demonstrated, based on the previous findings of pathological lacrimal drainage following anti-neoplasmic agents and exclusion of other etiologies, we report that T-DM1 can be a cause of lacrimal drainage system stenosis. It also shows that early diagnosis and treatment with topical steroid, which is more accessible and low-cost than surgical intervention, can reduce the burden of patients undergoing anti-cancer treatments.

Many studies have reported the adverse effects of chemotherapeutic agents on the lacrimal drainage system; however, those of targeted agents are less well known. Prompt diagnosis and treatment in the early phase of nasolacrimal obstruction can effectively decrease the burden of patients undergoing systemic anti-cancer treatments. Both oncologists and ophthalmologists need to be better informed of such adverse effects for prompt referral and effective early intervention.

## Data Availability

All data and materials are available in this article.

## Abbreviations

DM-1: Emtansine
FDDT: Fluorescein dye disappearance test
HER2: Human epidermal growth factor receptor 2
T-DM1: Trastuzumab emtansine
